# Lack of access to clean fuel and piped water and children’s educational outcomes in rural India

**DOI:** 10.1016/j.worlddev.2021.105535

**Published:** 2021-09

**Authors:** Pallavi Choudhuri, Sonalde Desai

**Affiliations:** a National Council of Applied Economic Research, New Delhi, India; b Department of Sociology, University of Maryland, 2112 Parren Mitchell Art-Sociology Building (Bldg. 146), 3834 Campus Drive, College Park, MD 20742, USA

**Keywords:** Clean fuel, Piped water, Maternal time-investment, Unpaid work, Children’s educational outcomes, Rural India

## Abstract

Investments in clean fuel and piped water are often recommended in developing countries on health grounds. This paper examines an alternative channel, the relationship between piped water and access to liquefied petroleum gas (LPG) and children’s educational outcomes. Results based on the second round of the India Human Development Survey (2011–12) for rural India show that children aged 6–14 years, living in households that rely on free collection of water and cooking fuel, have lower mathematics scores and benefit from lower educational expenditures than children living in households that do not collect water and fuel. Moreover, gender inequality in this unpaid work burden also matters. In households where the burden of collection is disproportionately borne by women, child outcomes are significantly lower, particularly for boys. The endogeneity of choice to collect or purchase water and cooking fuel are modeled via Heckman selection and the entropy balancing method.

## Highlights

• In addition to time-saving infrastructure, maternal time provides key inputs for children’s cognitive development.
• Children living in households that relying on free collection of water and firewood have lower educational outcomes.
• The negative impacts are greater when women bear a disproportionate burden of these unpaid activities.
• Gender inequality in unpaid work is associated with lower educational outcomes, particularly for boys.

## 1 Introduction

Time inputs and other parental endowments, especially those of the mother, provide important building blocks for children’s cognitive and non-cognitive development (Leibowitz, 1974, Flavio and James, 2009), and are crucial for later life outcomes (Becker, 1981, Heckman and Carneiro, 2003). The finite nature of time constrains the amount of time parents, can devote towards their children. Such constraints are more acutely felt when the mother is heavily involved in household chores, resulting from inadequate access to infrastructure, substituting time away from childcare and related activities. Two activities, that is, collecting firewood and fetching water, are particularly onerous in low- and middle-income countries. A large proportion of households in rural India rely on free collection of firewood and processing of dung cakes as well as fetching water from rivers and wells. Both these activities are predominantly undertaken by women, affecting the intra-household allocation of labor and the distribution of time allotment across other daily activities.

While the time burden of fetching water and firewood for women has been well recognized in the literature (Hirway & Jose, 2011), the impact of these activities on investments in children and children’s educational performance has received less attention. This paper attempts to bridge that gap in the literature.

Activities such as collecting fuelwood or fetching water are intended to supplement otherwise scarce household resources and improve the wellbeing of household members. Ease of collection of firewood and cost and ease of purchasing cooking fuel shape village energy market that may influence the decision regarding whether to rely on free collection of fuel or on its purchase. While fuelwood can be substituted by other forms of energy, water has no substitute, and access to water is often dependent on local infrastructure development. However, in both the cases, when such chores disproportionately affect women’s time allocation in domestic labor, it can substitute time away from engaging in productive or care activities. Such induced time poverty affects childcare activities in two ways. First, it may directly reduce the time available for supervising children and aiding their cognitive development. Second, it may induce children to join their parents in the drudgery of household activities such as cleaning, washing, or looking after siblings, thereby reducing the time available for educational pursuits.

In the context of the potential implications of women’s time use for their children’s education, in this paper, we use nationally representative 2011–12 data from the India Human Development Survey (IHDS) to examine the following questions:

a) Does access to the energy market (the ability to purchase LPG and firewood) and indoor piped water connections affect children’s educational outcomes?
b) In the absence of such household resources, how does the gender inequality inherent in the free collection of goods such as fuelwood or water affect children’s educational outcomes? Does this differ by the gender of the child?

The IHDS is unique in that it provides data not only on gendered differences in the time spent in paid or unpaid work, but also on educational outcomes. Additionally, it contains a range of other contextual variables, which facilitate the examination of how these correlate across household demographic characteristics, and village-level infrastructure facilities.

Part of the problem in exploring the synergy between the time burden of mothers and the educational outcomes of children lies in the difficulties of modelling this relationship, as family expenditure decisions are endogenous. Households with a higher aspiration to invest in children’s education may reduce other expenses and invest in purchasing fuel to ensure that mothers can spend more time in caring for children. Apart from budgetary allocations, evidence suggests that decisions on energy usage are also linked to the intra-household bargaining power of women (Choudhuri & Desai, 2020), and other socio-economic and cultural considerations (Lewis & Pattanayak, 2012). Endogeneity is less of a challenge in the case of access to piped water, which is largely driven by community-level water distribution systems, with the cost of installing piped water within the household premises being relatively small once such piped water network is available within the village or the community.

The rest of the paper is structured as follows: Section 2 discusses how inadequate access to clean fuel and indoor piped sources of drinking water influences women’s time allocation. Section 3 explains the synergy between the time investments of mothers and the educational outcomes of children. Section 4 presents the data and methodology, while Section 5 presents the results based on the findings of the paper. Section 6 delineates robustness checks, and Section 7, the concluding section, highlights the implications of the findings.

## 2 Resource dependence and time use

Existing literature (Bassani et al., 2010, Spears, 2012, Geruso and Spears, 2018) has shown that inadequate access to infrastructure, such as piped water, toilets, and clean fuel, can imperil health outcomes for children, which, in turn, can adversely affect children’s learning outcomes. Literature has also demonstrated that the use of solid fuels is closely related to higher levels of household air pollution and disease burden (Balakrishnan et al., 2011, Chafe et al., 2014, Gupta, 2019), which can act as serious impediments in children’s human capital development. This paper contributes to a somewhat different dimension of this nexus, that is, the relationship between access to infrastructure and children’s educational outcomes, mediated by a greater time burden on the mother (Crow & McPike, 2009).

### 2.1 Energy usage and time use

**Fig. 1:**
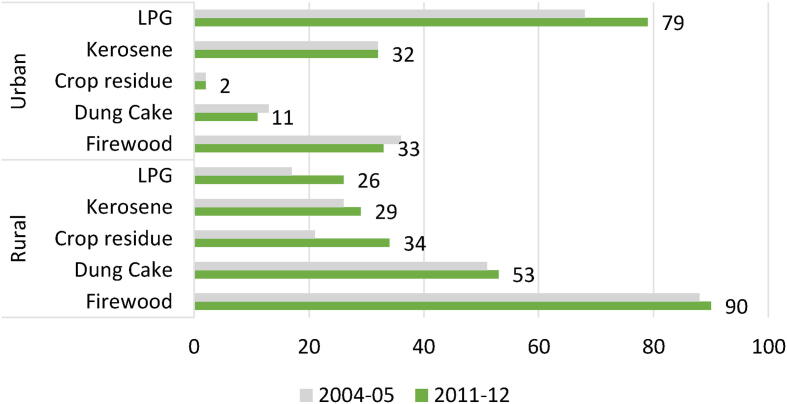
Patterns of Energy Usage by Households *Source:* Authors’ computation based on IHDS waves I and II. *Note*: Figures reflect population estimates for the household energy demand for cooking and heating.

IHDS data document that household energy usage patterns remained largely the same between 2004–05 and 2011–12, with a high dependence on traditional fuels such as firewood, dung, crop residue, and kerosene, as compared to more modern sources of fuel, such as LPG^1^ (see Fig. 1). The data also point to evidence of fuel-stacking behavior, with those who use LPG continuing to depend on common pool resources, such as firewood. Interestingly, for the task of collecting such biofuel for meeting their domestic energy needs is traditionally borne by women. If one considers the round-trip distance covered in minutes, including search time, this translates into women spending, on an average, more than two and half times the amount of time spent by men (see Table 1). For the purpose of this study, we define the energy market at the village level, as a household’s choice of fuel largely depends on local energy alternatives. This can be driven by access to common property resources or to infrastructure, and the rural livelihood structure, along with local cultural and socio-economic behavioral patterns (Choudhuri & Desai, 2020).

**Table 1:** Average Time (Minutes per Week) Spent on Activities by Households.

		Woman	Man
Fetching Water	Average time (minutes per week)	323	155
		(307)	(228)
	Participation rates ^(a)^	94.8%	70%
Collecting Firewood	Average time (minutes per week)	352	133
		(510)	(292)
	Participation rates ^(b)^	40.7%	25%

Source: Authors’ computation based on IHDS-II 2011–12.

*Note:* Figures in parentheses indicate standard deviations.

Participation rates refer to the proportion of households that report women/men involved in the respective unpaid activity over total households that (a) do not have access to indoor drinking water sources, and (b) households that collect fuelwood or straw from common property resources.

The *2012 World Development Report on Gender Equality and Development* (Bank, 2011) points out that one of the key benefits of energy interventions is the generation of a substantial amount of time savings, improved health, and better intra-household relations, along with greater scope for engagement in income-generating productive activities. However, the benefits accruing from energy intervention programs are realized only when such programs are aligned with local access to energy sources and household decision-making processes (Köhlin, Sills, Pattanayak, & Wilfong, 2011). The absence of such targeted intervention not only has implications for women’s time use, but also has the potential to trigger the intergenerational transmission of socio-economic disadvantages, including children’s educational outcomes.

### 2.2 Water and time use

**Fig. 2:**
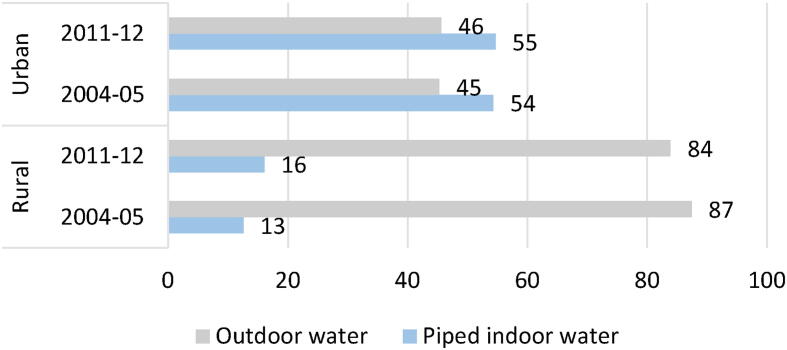
Indoor access to Piped Water *Source:* Authors’ computation based on IHDS, waves I and II. *Note*: Figures reflect population estimates of the percentage of rural households with access to piped drinking water.

Like the collection of biofuel such as firewood, fetching water from outside sources also imposes a substantial time burden on women. As per data from IHDS-II, while approximately 55 percent of urban households reported having access to piped indoor drinking water in 2011–12, only 16 percent of rural households had access to this facility (see Fig. 2). As many as 94.8 percent of the households without access to indoor water report women’s involvement in fetching water in the absence of indoor sources of water, compared to only 70 percent reporting male involvement. In terms of minutes spent, amongst the households that fetch water, women spend nearly double the time per week as compared to men in doing so (see Table 1)..

Using data from rural Pakistan, Ilahi and Grimard (2000) show that an increase in distance to the water source is positively associated with a higher proportion of women involved in fetching water, and negatively with labor-market engagement. In contrast, the construction of village water infrastructure and shared water taps in Kyrgyzstan resulted in significant time savings, reducing the time spent in home production (Meeks, 2017), while the acquisition of a piped water connection within the house led people in Morocco to spend more time in leisure and other social activities (Devoto, Duflo, Dupas, Parienté, & Pons, 2012).

There is evidence that not only does lack of access to piped water have implications for women’s own time use but that such access also improves a range of outcomes for children. Ravallion and Jalan (2003) found evidence of improvement in children’s health in rural India and Mangyo (2008), using panel data on China, showed that it had a positive effect on children’s health, albeit only in the case of more educated mothers. In a cross-country analysis involving nine developing countries, including India, Koolwal and Van de Walle (2013) noted that increased access to piped water not only reduces women’s unpaid work but also improves the extent of children’s enrolment in schools. However, there is scant evidence on the effect of availability of a piped water connection or adoption of clean energy on children’s educational outcome beyond enrolments.

## 3 Mother’s time investments and outcomes for children

Figures from 2011 to 12 IHDS data follow a trend that has been long observed over the decades, including data from the 1998–99 Indian Time Use Survey (TUS), conducted by NSSO, across the six States of Haryana, Madhya Pradesh, Gujarat, Odisha, Tamil Nadu, and Meghalaya (Hirway & Jose, 2011). The burden of such low-productivity non-market unpaid activities, coupled with the drudgery of multiple other household chores and care activities, leaves women with less time for producing marketable goods and services or for monitoring children’s activity. Left unsupervised, children’s schooling outcomes can get adversely affected, thereby providing a pathway for the transmission of an intergenerational disadvantage.

Mounting evidence suggests that maternal time influences children’s cognitive outcomes, with such outcomes helping build foundations for later life outcomes, such as improved earnings (Heckman et al., 2006, Carneiro et al., 2015) and health (Currie, Stabile, Manivong, & Roos, 2010). In the absence of detailed time use data mapped into children’s schooling outcomes, several studies have focused on maternal employment to measure the effect of mother’s time investments, though the results arrived at in these studies tend to be ambiguous. A section of the literature reports negative effects of maternal employment on child development (Bernal, 2008, Hill et al., 2005), but other studies suggest that such employment does not reduce time investments on children, with mothers often reducing leisure time (Bianchi, 2000), (Sayer, Bianchi, & Robinson, 2004), and in some instances mothers who are not employed may still not be able to devote time to children due to other domestic demands (Desai & Jain, 1994). Ruhm (2008) further demonstrated that maternal employment benefits children from relatively disadvantaged backgrounds, but such benefits wane for children who are better off, and in fact, have an adverse effect due to the substitution effect of mother’s labor supply on the time she could have spent at home (Desai, Chase-Lansdale, & Michael, 1989).

More recent studies in the context of developing countries, such as India, suggest that the relationship between maternal employment and children’s outcome is non-linear, and varies across the type and intensity of employment and educational attainment of the mother. Using data from IHDS wave I (2004–05), Vikram, Chen, and Desai (2018) find that children of salaried mothers with lower levels of education are likely to suffer from disadvantages due to time constraints posed by the time away from home spent by mothers. This inverse relationship flips at higher levels of maternal education, indicating positive returns to education, with gains in financial resources potentially improving children’s educational outcomes. In contrast, not sufficient evidence is available on the effect of time investment in unpaid and low-productivity chores such as the free collection of goods on child outcomes.

Women’s intensive engagement in unpaid work for household production, such as cooking, cleaning, fetching water, and numerous such activities, may come at the cost of foregoing engagement in regular jobs with better pay, with consequent negative spill-over effects on their children through the income effect. The negative spill-over effect gets enhanced further through the substitution effect if such engagement in unpaid activities is particularly time-intensive, substituting time away from childcare. Kabeer (2012), for instance, discusses how women in rural areas, who invest long hours in domestic activity, are subject to intense time poverty.

Primitive models of household theory, such as the unitary model or the common preferences model, suggest that all household members behave as a single unit or that decision-making rests with a benign dictator (Becker, 1993). But, more recent research in intra-household bargaining models suggests that men and women have different expenditure preferences and do not necessarily pool their resources (Agarwal, 1997, Lundberg et al., 1997, Seiz, 1995, Woolley, 1993). This has substantial implications for investments in children, especially on breaking the transmission of an intergenerational gendered disadvantage. The literature indicates that when women have greater access to resources, they are more inclined to invest in children as compared to men, especially in daughters (Menon, Van Der Meulen Rodgers, & Nguyen, 2014).

Women’s participation in labor markets, brings with it maternal absence but also additional income. This may increase investments in children through enrollment in higher quality private schools (Muralidharan and Kremer, 2008, Desai et al., 2009), or greater expenditure on books and school materials. Alternatively, increased access to financial resources can pave the way for housekeeping services, allowing for greater flexibility of time for the mother. In particular, women’s participation in the labor market, at higher levels of maternal education, improves children’s wellbeing, including their cognitive development (Doepke & Tertilt, 2011).

In contrast, hand, women’s time away from home for carrying out non-market unpaid tasks, and their subsequent inability to tap into the labor market may result in both maternal employment and time spent on caring for and teaching children.

The demands on parents’ time towards children’s education have been increasing over time, with children being tasked nearly on a daily basis with carrying out at-home assignments based on the school curriculum, which require parental supervision (Vikram et al., 2018). Hence, time demands for free collection of fuel and water may affect children’s schooling outcomes by reducing the time spent by unsupervised children on the assigned tasks from school.

Another, but related, channel that can sow the seeds of an intergenerational disadvantage is through learning outcomes. The lack of maternal supervision can adversely affect children’s cognitive development. This can get exacerbated for mothers who have to routinely spend time away from home. While spending time away for income-generating activities adds to the pool of household income and can still have a net positive effect on learning outcomes with the availability of resources, we do not expect such positive transmission on learning with involvement in low-productivity unpaid chores.

Based on the argument presented so far, we propose the following hypotheses:

Hypothesis H1: Access to clean and time-saving sources of energy and indoor piped sources of drinking water are expected to improve children’s educational outcomes.

Hypothesis H2: When this burden of free collection of fuel and water falls disproportionately on women, children’s educational outcomes are further depressed.

Hypothesis H3: In the absence of clean time-saving energy sources or piped water access, we expect girls to be more adversely affected than boys with increase in mother’s share of time invested in fetching water or collecting firewood

## 4 Data and methodology

This study uses the nationally representative sample from wave II of the India Human Development Survey (IHDS), 2011–12 (Desai & Vanneman, 2018)^2^. The sample comprises 23,439 children aged 6 to 14 years from 13,187 rural households across 1452 villages. These households are spread across the 33 States and Union Territories of the country, with the exception of the Andaman and Nicobar Islands and Lakshadweep. In contrast to regular time use data, the IHDS provides data not only on the gendered differences in time spent in paid versus unpaid work, and children’s study time, but also on cognitive attainment, though the latter is only available for a subset of the population, that is, for children aged 8–11 years. Additionally, IHDS contains a range of other contextual variables, which enables examination of their correlation across demographic characteristics of households and village-level infrastructure facilities.

### 4.1 Dependent variables

We use the following four alternate measures of children’s educational outcomes:

(i) *Study time*: We compute children’s study time as the total time spent by the enrolled children, aged 6–14 years, measured in terms of the minutes per week. To take into account the time spent at home on assigned school work (such as homework assignments), we further add the time spent on homework per week, measured in minutes.
(ii) *Annual educational expenses*: Our second indicator includes school fees, expenses on books and uniform, and transportation cost incurred over the last one year.
(iii) *Math score*: This indicator was captured for only a subset of the population, that is, for children aged 8–11 years. The test scores for arithmetic skills were recorded across the following four categories: (a) cannot read numbers above 10, (b) can read numbers between 10 and 99, (c) can subtract a two-digit number from another, and (d) can divide a number between 100 and 999 by another number between 1 and 9. For our study, we construct a binary variable, with the student assigned a value of one if she/he can subtract or divide, and zero otherwise.

On an average, children spent approximately 32.5 h in school per week, with another 7.6 h on homework assignments. Further, annual educational expenses amounted to approximately INR 2372 on an average, with school fees comprising nearly 47.6 percent of the expenditure. These expenses differ across the type of schools, with the annual expenses incurred for private schools being seven times more than those incurred for government or government-aided schools for the sample under consideration. For the subset of the population that took the math test, it was found that nearly 20 percent could not identify numbers, while another 39 percent could only recognize numbers. Only 15 percent could carry out both subtraction and division.

### 4.2 Share of maternal time in unpaid work

To test hypothesis H2, we define the share of maternal time spent in unpaid work as the ratio of the time spent by the mother on the respective unpaid work under consideration to the total time spent on the task by both the parents. IHDS provides this data for collecting fuelwood and fetching water for adult males and adult females within the family, which we use as a proxy for the father’s time and the mother’s time spent on the activity. We use the share of the time spent by adult women at the household level, which allows us to capture within-household gender-based heterogeneity in time endowments in rural areas. Based on the scenarios under consideration in this paper, we use the following two alternate measures of unpaid work:

(i) Time spent in collecting firewood—this includes travel time, to and fro, along with search time for collection, measured in minutes per week.
(ii) Time spent in fetching water—this comprises the time spent on traveling, including the wait time for fetching water, measured in minutes per week.

Table 2 presents the descriptive statistics of the variables used for our analysis in the context of energy needs and access to indoor piped water.

**Table 2:** Sample Statistics.

Variables	Mean	Standard Deviation
Distance to school (in kilometers)	2.04	3.32
Children’s standard in school	4.6	2.6
Annual household income (INR)	93030.7	147570.2
*Percent of Households:*		
Gender of the child (Female)	48	
Enrolled in government school	72.50	
Household has electricity	73.77	
Mother’s education:		
Illiterate (omitted)	49.64	
Primary (1–5)	16.88	
Middle (6–9)	19.81	
Secondary (10–11)	6.51	
Higher secondary + (12–14)	4.37	
College degree and above (15+)	2.78	
Social Groups		
Upper Caste (Omitted)	15.14	
Other Backward Class (OBC)	38.17	
Scheduled Caste	23.77	
Scheduled Tribe	9.2	
Muslim	12.79	
Christian, Jain, Sikh	0.96	
Place of residence: less developed village	58.06	
More developed (omitted category)		
Sample size (unweighted)	23.439	

*Source:* Authors’ computation based on IHDS-II data, 2011–12.

*Note:* Observations have been weighted to reflect the 2011 Indian population.

### 4.3 Decision to collect firewood

Family expenditure decisions on adopting clean fuel energy for cooking are likely to be endogenous—households with a higher aspiration to invest in children’s education may reduce other expenses and invest in purchasing clean fuel to ensure that mothers can spend more time in childcare and related activities. We use village-level price data to model this source of endogeneity. To account for the possibility of household level self-selection in collecting firewood versus adopting other sources of energy, we follow a Heckman-control function approach, also known as Heckman selection model (Heckman, 1976), using maximum likelihood estimation. Accordingly, we consider the following underlying model:

(1) $$Y_{i}=\alpha +\beta X_{i}+\gamma Z_{i}+\varepsilon_{i}$$

where, $Y_{i}$ = alternate measures of children’s educational outcome captured by the study time, educational expenses, and Math test scores. For the analysis involving the Math score, we use the Heckman-control function adjusted Probit model, as our dependent variable in the outcome equation is a binary variable.. $X_{i}$ is a vector of explanatory variables for the *i*-^th^ household. These include child-level controls, such as children’s gender and standard in school; school-level controls: type of school (public or private), distance to the school (in kilometers); the share of maternal time in unpaid work (that is, collecting fuelwood); household-level characteristics, such as caste/religious background of the parents, highest level of education obtained by the mother in the household, whether the household uses electricity, and annual household income. States are the locus of the structure of education and several other policies, and this varies from state to state — we use state dummies to capture time-invariant state-level unobservables and other governance and policy initiatives and their effectiveness. We also control for the level of development in a village, that is, whether it is less or more developed.

$Z_{i}$, controls for household level selectivity for collecting fuelwood. Measured as a binary variable, $Z_{i}$ takes the value of one for households that collect firewood and zero otherwise. Using Heckman control function, we further define the model for household-level self-selection in Equation (2), bringing in the factors that could affect the household’s decision on collecting fuelwood. The model for household self-selection for fuelwood collection from the commons is defined as follows:

(2) $$Z_{i}=\lambda +\delta W_{i}+{∊}_{i}$$

where, $W_{i}$ indicates the set of instruments. α, β, γ, λ, and δ in Equations (1), (2) are parameters to estimate. $\varepsilon_{i}$ and ${∊}_{i}$ are the unobserved random error terms. Equations (1), (2) are jointly estimated following the maximum likelihood approach.

Household-level self-selection is based on household energy demand, which depends on access (physical access and price) to the sources of energy, along with other socio-economic characteristics. The selection equation is identified by instruments that affect household-level decisions on energy usage, such as the village-level market price of firewood, the village-level price of LPG, the type of village road, and the ratio of female to male wage rate for unskilled labor at the level of a village. We argue that while these factors are likely to affect a household’s decision to collect fuelwood, they are exogenous to the households, and unlikely to directly affect household-level inputs for children’s educational outcomes.

The first two identifying instruments capture the effect of the market price of energy products on household behavior. The village population is small enough that households are unlikely to hold any market power over setting the price of firewood or LPG. While the price of LPG is determined by state-run oil companies, transportation and distribution costs at the local level induce price differentials across villages and towns, making price external to the household. The average price of LPG cylinders is INR 443 in the sample under consideration, with a standard deviation of 63.66, but we observe variation not only across states, but also within states—the within-state standard deviation of price ranges from INR 13.33 in Uttarakhand to INR 154.6 in Jharkhand. Low-income households are likely to be particularly sensitive to the higher price of LPG, and may opt for the time-intensive unpaid activity of collecting firewood. Interestingly, evidence suggests that households in the upper income quintiles continue to use biofuel, guided by local access to alternate sources of energy, or tastes and preferences, (Narain, Gupta, & Van't Veld, 2008), (Lewis & Pattanayak, 2012), or intra-household bargaining power (Gould and Urpelainen, 2019, Choudhuri and Desai, 2020).

As in the case of the LPG price, when the price of firewood rises, households are more likely to switch towards collecting fuelwood that is available for free from the village commons. Based on a study in Nepal’s Terai region, Amacher, Hyde, and Kanel (1996) found that households are sensitive to the price of firewood, spending more time in collecting firewood when there is an increase in its market price.

Our third identifying instrument is the ratio of female to male wage rate, capturing opportunities for women for unskilled wage labor. Amacher et al. (1996) found evidence that the household decision to collect firewood is guided by alternate labor opportunities, indicating that as the remuneration from such work goes up, the opportunity cost of spending time in collecting firewood also increases. A higher wage ratio will be negatively associated with the collection decision. An increase in the income-generating capacity of women enhances the purchasing power of households, thus providing households with wider access to alternate energy markets.

Other factors that can influence the household-level decision to collect firewood, as well as children’s learning and the time spent by them in school work, are whether the household uses electricity, caste and religion groups, and annual household income. Villages across rural India differ in terms of access to health facilities, connectivity to road networks and towns, and other infrastructure facilities. Based on a list of ten infrastructure facilities, villages have been classified as per their level of development. Villages have been considered as developed if they have at least six out of the following ten facilities: electricity, paved road, grocery/*kirana* shop, bus stop, telephone access (landline and mobile), post office, police station, market place/*bazaar*, and bank branches. Village-level infrastructure can provide indications of physical access to energy markets, providing households with greater flexibility in adopting clean energy such as LPG. Since these factors also affect the type of education children receive, though we control for them, we do not treat them as instruments for the Heckman control function. We also include state dummies to account for time-invariant state-level unobserved characteristics.

### 4.4 Decision to fetch water

Endogeneity is less of a challenge for piped water, as the cost of installing a piped water system, once such a network is available in the village, is relatively small. Data from IHDS-II (2011–12) show that 41 percent of rural households have access to indoor water, of which only 38 percent (16 percent of all rural households) have access to piped drinking water, with another 36 percent and 14 percent using hand pumps and tubewells, respectively. While richer households are more likely to have indoor water access, the survey data reveal that nearly 43 percent and 25 percent of the top two income quintiles (fourth and fifth quintiles, respectively) do not have indoor sources of water, and fetch water from outside from community water taps, tubewells, open wells, hand pumps, or natural sources of water. This emphasizes the role of village infrastructure and water systems, and the role of local administrative units in facilitating access to drinking water, which are largely external to the households. In the absence of access to indoor drinking water, who fetches water is often guided by intra-household bargaining power, customs, community practices, gendered division of labor, and the relative valuation of women’s time in domestic labor and market-based work (Doss, 2013).

Across developing nations, evidence suggests that women are typically more involved in household labor and are deemed as the primary water-bearers (Ray, 2007), with adverse implications for health, human capital development, and labor force participation (Jayachandran, 2015, Geruso and Spears, 2018), including intergenerational disadvantage.. Kookana et al. (2016), using data from two watersheds in the semi-arid regions of Rajasthan and Gujarat, find evidence of school absenteeism linked to groundwater scarcity, with girls missing out on school more frequently than boys.

To account for between-community effect or village-level variation in the water distribution network and related infrastructure, and its potential effect on household behavior, we adopt a hierarchical analysis for this section, using multi-level random effects regression (Goldstein, 2011, Raudenbush and Bryk, 2002), represented in Equation (3), with individuals in households nested within their village of residence. This allows us to model the effect of neighborhood disadvantage, and how it contributes to children’s outcome. With only 16 percent of all rural households reporting indoor access to piped drinking water, the role of village infrastructure assumes a critical role in providing access to piped water supply and in intergenerational transmission of disadvantages amongst those without access to piped water.

The analysis for this section is represented by the following multi-level random-intercept model:

(3) $$Y_{\mathrm{ij}}=\alpha +\beta X_{\mathrm{ij}}+\varepsilon_{\mathrm{ij}}+\nu_{j}$$

where, $Y_{\mathrm{ij}}$ indicates our set of dependent variables for child *i* nested within cluster *j*, with each village representing a separate cluster. $X_{\mathrm{ij}}$ are the covariates comprising child-level variables (standard in school, gender), school-level variables (distance to the school and whether the school is government-funded or private), mother’s characteristics (education level, share of time spent in fetching water), and household-level variables (household income and number of persons). $\varepsilon_{\mathrm{ij}}$ is the level one residual defined at the individual level; $\nu_{j}$ is the random effect for each cluster, defined at the village level. We also control for state fixed effects to take into account differences in education policy that typically vary from state to state. We drop the States of Bihar and Assam from our study, as these two states have an extremely small proportion of rural children living in households with indoor piped water connections, leading to a highly skewed distribution of control (without indoor connection) versus treated (with piped water connections) groups for the sample under consideration.

The random effects, $\varepsilon_{\mathrm{ij}}$ and $\nu_{j}$, are assumed to follow normal distributions with zero mean, and variances σ and $\phi_{0}$ respectively. The associated intra-village correlation, measuring intra-class correlation (ICC), provides an estimate of the role of within-village clustering, and is defined as the ratio of between-village (level two) variance over the total variance (measured as the sum of the estimated level two and level one variance), ${\mathrm{ICC}}_{1}=\frac{\phi_{0}}{\phi_{0}+\sigma }$. For our model where we use Math scores for our dependent variable, we estimate multi-level Probit models, with the ICC defined as ${\mathrm{ICC}}_{2}=\frac{\phi_{0}}{\phi_{0}+\frac{\pi^{2}}{3}}$, with π is measured as 3.14159.

While the above methodology allows us to examine how village-level random effects are associated with schooling outcomes, after controlling for a set of covariates as well as maternal time in unpaid work, we cannot comment on causality. As with any observational data, it is difficult to rule out potential endogeneity arising from household location or expenditure decisions, with the possibility that households without access to indoor water are systematically different from those that do have such access. We observe that 92.5 percent of the households which report indoor access to piped drinking water reside in villages that have piped water. However, only 36.8 percent of the households in such villages have within-household access. This essentially means that if we consider indoor access to piped water as a treatment variable, confoundedness could affect both the treatment status and the potential outcome, preventing us from commenting on the extent of improvement in our educational outcomes resulting from the treatment.

We define $Y_{1}$ and $Y_{0}$ as the outcomes of the treated and non-treated samples, respectively, with T, a binary treatment variable equal to one if the household has access to an indoor piped water connection (treated), and zero otherwise (not treated). The observed outcome for child *i* is measured as $y_{i}=Ty_{i1}+\left(1-T\right)y_{i0}$. Our interest lies in estimating the Average Treatment Effect on the Treated (ATT), which measures the difference between the expected schooling outcome of children for those with access to piped water versus the counterfactual outcome, that is, if these children did not have access to piped water. ATT is expressed as:

(4) $$ATT=E\left[Y_{1}-Y_{0}|T=1\right]=E\left[Y_{1}|T=1\right]-E\left[Y_{0}|T=1\right]$$

However, the potential outcome, $Y_{1}$ is never realized in the treatment state as the counterfactual E[$Y_{0}$|T = 1], is unobservable, and needs to be estimated. Identification is achieved if the assumption of conditional independence holds—if once we control for observables, the decision to install piped water connection can be treated as random.

One of the matching techniques, which gets used often for non-experimental studies, is Propensity Score Matching (PSM), introduced by Rosenbaum and Rubin (1985). Through the use of PSM, control units are matched to the treated units based on estimated treatment probabilities, termed as propensity scores, correcting for any sample selection bias. However, estimating PSM often comes at the cost of discarding units for which balance is not achieved, thereby reducing the sample size. In contrast, the entropy balancing (EB) method, developed by Hainmueller (2012), allows the user to exploit the full sample for carrying out the analysis, assigning a scalar weight to each individual unit in the control group, based on a set of balance constraints, while keeping the weights close to the base weights—this prevents any of loss of information for regression analysis in the next step. In effect, EB assigns a weight of one to the treated units, and re-weights all individual units in the control group to ensure balance with those in the treated group, with the weights defined in terms of the pre-specified sample moments of the covariate distribution, such as means, variances, and skewness. These re-weighted units are then used for estimating the ATT.

Following re-weighting, the treatment becomes moment-independent of control variables, reducing the unobserved variance in our outcome. This allows us to estimate the counterfactual mean in Equation (4) as follows:

$$E\left[\hat{Y_{0}}|T=1\right]=\frac{\sum_{i|T=0}Y_{i}\omega_{i}}{\sum_{i|T=0}\omega_{i}}$$

where $\omega_{i}$ is the weight assigned to each control unit based on the re-weighting mechanism that minimizes the entropy distance metric subject to the balance constraints imposed on the covariate moments of the control group. In the next step, we estimate the effect of our treatment variable, indoor piped water connection, on the re-weighted sample, controlling for a set of observables (employed for EB), using the Ordinary Least Squares (for study time and educational expenses) and Probit (Math test score) approach, respectively. The list of covariates used for entropy balancing, along with the quality of balancing, is provided in Section 5, followed by results from the regression analysis using the balanced sample.

## 5 Results

### 5.1 Energy needs and outcomes for children

Table 3A, Table 3B present estimates for all selection and outcome equations respectively. Estimates from the first stage selection Equation (2) indicate that households in more developed villages are less likely to collect fuelwood. Also, the probability of collection reduces with an increase in household income. We further observe that both the market prices of LPG and firewood are positively linked to the household’s decision to collect fuelwood, though only the price of LPG is statistically significant (see Table 3A). For a 10 percent increase in the median village-level market price of an LPG cylinder, priced at INR 450, the estimated marginal effects show that the probability of collecting fuelwood increases by 1.8 percentage points. The coefficient for the ratio of the female to male wage rate for unskilled labor is negative, but not statistically significant.^3^

**Table 3A:** Factors Determining Household Fuelwood Collection (First Stage Results).

Fuelwood Collection	Study Time	Educational Expenses	Math Score
	I	II	III
Household has electricity	−0.38***	−0.34***	−0.39***
	(0.06)	(0.03)	(0.06)
Caste and Religion (*Ref:* Forward Caste Hindus 1)			
OBC 2	0.02	−0.01	0.11
	(0.07)	(0.07)	(0.08)
Scheduled Caste 3	0.13*	0.10	0.16*
	(0.07)	(0.07)	(0.08)
Scheduled Tribe 4	0.37***	0.31***	0.44***
	(0.09)	(0.09)	(0.11)
Muslim 5	−0.18*	−0.23**	−0.11
	(0.10)	(0.09)	(0.11)
Christian, Sikh, Jain 6	0.07	0.05	0.09
	(0.17)	(0.18)	(0.22)
Village: more developed 1	−0.22***	−0.23***	−0.19***
*Ref:* Less developed 0	(0.06)	(0.06)	(0.07)
Annual household income (log)	−0.10***	−0.09***	−0.13***
	(0.02)	(0.02)	(0.03)
Female to male wage ratio	−0.24	−0.25	−0.24
	(0.19)	(0.17)	(0.22)
LPG price (log)	0.63***	0.63***	0.85***
	(0.17)	(0.16)	(0.24)
Fuelwood price (log)	0.00	0.02	0.02
	(0.03)	(0.03)	(0.03)
Constant	−1.48	−1.60	−2.52*
	(1.05)	(1.00)	(1.49)
State Dummies	Yes	Yes	Yes
Observations	15,664	15,191	5553
Wald test of indep. eqns.(rho = 0): chi2(1)	6.44	98.76	7.20
Prob > chi2	(0.01)	(0.00)	(0.01)

*Source:* Authors’ computation based on data from IHDS-II (2011–12).

*Note:*^##^Column III reports Wald statistics with chi squared (31).

First stage results from the Heckman Selection model correcting for fuelwood collection by the household. The estimates reflect Probit coefficients. The dependent variable is whether the household collects fuelwood from the village commons. The results have been estimated jointly with the outcome equation using the Maximum Likelihood approach. Standard errors are clustered at the level of the primary sampling units (villages); reported in parentheses. *** p < 0.01, ** p < 0.05, * p < 0.1.

**Table 3B:** Effect of Fuelwood Collection on Children’s Schooling Outcomes (Second Stage Results).

Variables	Study Time	Educational Expenses	Math Score
	I	II	III
School type (private = 1)	−0.07***	−1.72***	−0.47***
*Ref*: government = 0	(0.01)	(0.04)	(0.08)
Distance to school	−0.01***	0.06***	−0.00
	(0.00)	(0.01)	(0.01)
Household has electricity	0.02	0.30***	0.13*
	(0.01)	(0.04)	(0.07)
Mother’s education (*Ref:* Illiterate 1)			
1–4 std.	0.02	−0.00	0.20***
	(0.01)	(0.03)	(0.07)
5–9 std.	0.04***	0.10***	0.27***
	(0.02)	(0.03)	(0.07)
10–11 std.	0.02	0.15**	0.43***
	(0.03)	(0.06)	(0.10)
12th & some college	0.03	0.36***	0.59***
	(0.03)	(0.06)	(0.13)
Graduate & above	0.08***	0.30***	0.35**
	(0.03)	(0.07)	(0.17)
Caste and Religion (*Ref:* Forward Caste Hindus 1)			
OBC 2	0.03	−0.07***	0.19***
	(0.02)	(0.03)	(0.07)
Scheduled Caste 3	0.02	−0.17***	−0.08
	(0.02)	(0.03)	(0.08)
Scheduled Tribe 4	0.01	−0.35***	−0.19**
	(0.03)	(0.04)	(0.09)
Muslim 5	−0.03	−0.27***	−0.21**
	(0.03)	(0.04)	(0.10)
Christian, Sikh, Jain 6	0.01	0.31***	−0.05
	(0.03)	(0.10)	(0.27)
Village: more developed 1	−0.02	0.11***	−0.05
*Ref:* Less developed 0	(0.02)	(0.02)	(0.05)
Gender: Girl 1	−0.02*	−0.09***	−0.15***
*Ref:* Boy 0	(0.01)	(0.02)	(0.06)
Share of mother’s time in fetching fuelwood	−0.05***	−0.10***	−0.12*
	(0.02)	(0.03)	(0.07)
Girl*Share of mother’s time	0.03*	0.04	0.00
	(0.02)	(0.04)	(0.10)
Annual household income (log)	0.00	0.10***	0.02
	(0.01)	(0.01)	(0.03)
Constant	7.68***	7.52***	−1.14**
	(0.10)	(0.15)	(0.46)
Standard in school (dummies)	Yes	Yes	Yes
State Dummies	Yes	Yes	Yes
Wald chi^2^	453.06	16861.90	560.38
Prob > chi2	0.00	0.00	0.00
Number of observations	15,664	15,191	5,553

*Source:* Author’s computation based on data from IHDS-II (2011–12).

*Note:* The results are from the outcome equation from the Heckman Selection Model, correcting for fuelwood collection by the household. Estimates in Column III reflect Probit coefficients. The results have been estimated jointly with the selection equation, using the Maximum Likelihood approach. Standard errors are clustered at the level of primary sampling units (villages); reported in parentheses. *** p < 0.01, ** p < 0.05, * p < 0.1.

Across all models, we observe that enrollment in public/government schools translates into lesser time spent on studies, lower educational expenses, and lower Math score (see Table 3B). While school fees are likely to be lower in public schools, because of state education policies, a lower score in Math test and less time spent in school and on homework assignments may speak to the poorer quality of education in such schools, something also pointed out in prior research (Desai et al., 2009).

Analyzing the predicted values of the outcome variables from the Heckman selection model (Equation (1)), we find that the schooling outcomes are lower amongst households that collect firewood as compared to households that do not. The probability of being able to subtract or divide decreases for children in households that collect fuelwood by approximately 6–7 percentage points. Further, girls suffer from a systematic disadvantage across all three schooling outcomes, though the effect is more pronounced in terms of educational expenses and Math test scores. What is even more striking from these estimates presented in Table 3C is the extent of the gender-based differential—the mean predicted probability of being able to subtract or divide for girls, when they are part of households that do not collect, is at the same level as the mean predicted probability for boys, who are part of households that do collect, at 0.38 percentage points.

**Table 3C:** Predicted Value (mean) of Children’s Schooling Outcomes, Correcting for Self-selection.

		Study Time (log)	Educational Expenses (log)	Math Score (probability)
		I	II	III
Girls	Collects fuelwood	7.73	6.57	0.31
	Does not collect	7.72	6.83	0.38
Boys	Collects fuelwood	7.74	6.82	0.38
	Does not collect	7.75	7.14	0.44

*Source:* Author’s computation based on data from IHDS-II (2011–12).

*Note:* the results are from the outcome equation from the Heckman Selection model, based on estimates from Table 3B.

**Fig. 3:**
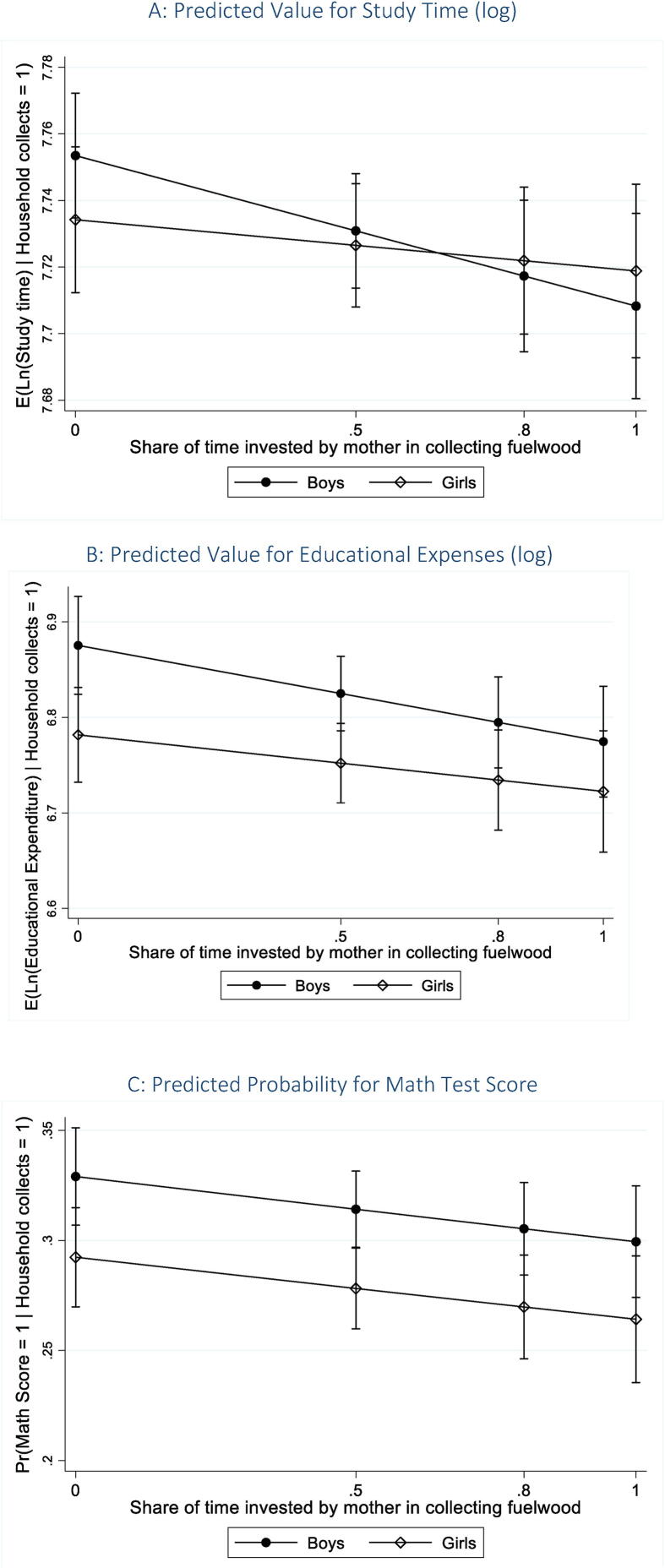
Effect of Mother’s Share of Time Invested in Collecting Fuelwood. *Source:* Authors’ computation based on data from IHDS-II (2011–12). *Notes:* Predicted values for children’s outcome at different values of the ratio of the time invested by the mother to the total time invested by both the parents in collecting fuelwood (0, 25th percentile, 50th percentile, 75th percentile). These predictive margins are based on estimates provided in Table 3B, using Heckman regression, correcting for self-selection.

Conditional on households collecting fuelwood, the results from Table 3D also indicate that gender inequality in responsibilities for unpaid activities has a negative impact on child outcomes. We interact the gender of the child with the ratio of the mother’s time to the total time invested by both the parents in collecting fuelwood—this allows us to examine our hypothesis on intergenerational disadvantage being transmitted along gendered lines. Our results indicate that the marginal effect of an increase in the mother’s share of time invested in unpaid work on children’s study time, is (-)0.05 for boys and (-)0.02 for girls (Table 3D, column I); the effect is, however, statistically significant (at the one percent level) only for boys, with the p-value of the co-efficient for girls at 0.226. Fig. 3A shows the predicted values at different levels of the share of mother’s time invested—at 0.5, 0.8, and 1^4^, which are the 25th, 50th (median), and the 75th percentiles, respectively. Our findings suggest that while the predicted values of children’s study time are higher for boys at relatively low levels of the mother’s share of unpaid work. In contrast, at the median value of the ratio and beyond, the predicted values are lesser for boys, refuting our hypothesis (H3). It is quite likely that as mothers spend more time away from home towards collecting fuelwood, they have less time to spend towards monitoring their children—this adversely affects boys more than girls.

**Table 3D:** Marginal Effect of a Change in the Share of Mother’s Time in Collecting Fuelwood.

	Study Time (log)	Educational Expenses (log)	Math Score(Probability = 1)
	I	II	III
Boys	−0.05***	−0.10***	−0.03*
	(0.02)	(0.04)	(0.02)
Girls	−0.02	−0.06	−0.03
	(0.02)	(0.04)	(0.02)

*Source:* Author’s computation based on results from Table 3B, using data from IHDS-II (2011–12).

*Note:* The coefficients reflect the marginal effect of a change in the share of mother’s time in collecting fuelwood out of the total time invested by the parents towards the task. Standard errors have been calculated using the delta method. *** p < 0.01, ** p < 0.05, * p < 0.1.

In terms of annual educational expenses, the results suggest that the adverse effect from an increase in the share of mother’s time in collecting fuelwood persists. Disaggregating by the gender of the child, we observe that the adverse effect is nearly twice as high for boys in response to an increase in the mother’s share of time invested in unpaid work, though the marginal effect is statistically significant for only boys (Table 3D, column II). Fig. 3B shows the predicted values at different levels of the share of mother’s time invested—while the effect of an increase in the mother’s share of unpaid work on educational expenses is negative for both boys and girls, the rate of decrease is higher for boys with increases in the share variable. It is likely that as the mother’s share of the time invested in unpaid work away from home increases, this affects the educational expenditure through the substitution effect, with fewer resources at the disposal of the family to invest in children’s education. Prior research, as discussed in Section 3, suggests that an increase in mother’s financial resources improves children’s outcome. However, as the time invested in the drudgery of unpaid work goes up, the mother is less likely to be able to invest her time towards more productive income-generating activities.

We carry out our analysis on children’s Math test score for a subset of the IHDS-II sample aged 8 to 11 years—approximately 8,224 children residing in rural India. After the missing observations across variables are left out, the sample size is reduced to 5,553 children. The results show that after controlling for other factors, the marginal effect of an increase in the share of the mother’s time decreases the probability of scoring on the Math test, but the effect is statistically significant only for boys, with a p-value of 0.075 (Table 3D, Column III). Fig. 3C shows the predicted probabilities at different levels of the share of time invested by the mother.

### 5.2 Piped water access and children’s outcome

For examining the effect of access to piped water on children’s outcomes, we carry out a multi-level regression analysis, with individual children as the level one units, nested within villages, which are our level two units. The results from the multi-level analysis allow us to measure the variation in schooling outcomes attributable to villages, after controlling for all explanatory variables. The intra-class correlation (ICC), measuring the proportion of between village variation to the total variation is 0.40 for model I, indicating that 40 percent of the variation in children’s study time is between villages, while 60 percent of the variation is between children residing within the same village. The between-village variation is less for educational expenses (Model II) and the Math test score (Model III) at 27 percent and 7.24 percent respectively.

The fixed part of the model shows expected direction of the relationship between the correlates and our outcome variable (see Table 4A). Enrollment in private school improves all three educational outcomes, consistent with prior studies (Desai et al., 2009). Access to resources matters, and household-level characteristics, such as the annual household income, or the presence of electricity, improves children’s schooling outcomes.

**Table 4A:** Effect of Indoor Piped Water Connection on Children’s Schooling Outcomes.

Variables	Study Time	Educational Expenses	Math Score
	I	II	III
School type	−0.06***	−1.74***	−0.52***
	(0.01)	(0.02)	(0.05)
Distance to school	−0.00***	0.05***	0.01
	(0.00)	(0.00)	(0.01)
Household has electricity	0.04***	0.15***	0.20***
	(0.01)	(0.02)	(0.06)
Mother’s education (*Ref:* Illiterate 1)			
1–4 std.	0.01	0.03	0.18***
	(0.01)	(0.02)	(0.05)
5–9 std.	0.01*	0.16***	0.38***
	(0.01)	(0.02)	(0.05)
10–11 std.	0.03**	0.27***	0.53***
	(0.01)	(0.03)	(0.08)
12th and some college	0.01	0.40***	0.67***
	(0.01)	(0.03)	(0.10)
Graduate and above	0.06***	0.40***	0.44***
	(0.02)	(0.04)	(0.12)
Caste and Religion (*Ref:* Forward Caste Hindus 1)			
OBC 2	−0.00	−0.09***	0.00
	(0.01)	(0.02)	(0.06)
Scheduled Caste 3	−0.01	−0.21***	−0.21***
	(0.01)	(0.02)	(0.06)
Scheduled Tribe 4	−0.01	−0.23***	−0.29***
	(0.01)	(0.03)	(0.09)
Muslim 5	0.01	−0.26***	−0.18**
	(0.01)	(0.03)	(0.08)
Christian, Sikh, Jain 6	−0.01	0.24***	−0.29
	(0.03)	(0.06)	(0.19)
Village: more developed 1	0.01	0.06**	−0.01
*Ref:* Less developed 0	(0.02)	(0.03)	(0.05)
Gender: Girl 1	0.01	−0.08***	−0.19***
*Ref:* Boy 0	(0.01)	(0.02)	(0.05)
Share of mother’s time in fetching water	0.01	−0.10***	−0.13*
	(0.01)	(0.03)	(0.07)
Girl*Mother’s share of time	−0.01	0.01	0.04
	(0.01)	(0.03)	(0.09)
Household income (log)	0.01**	0.06***	0.05**
	(0.00)	(0.01)	(0.02)
Constant	7.65***	7.52***	
	(0.07)	(0.14)	
Indoor piped water 1	0.01	0.03	0.00
*Ref:* No indoor piped water 0	(0.01)	(0.02)	(0.06)
Standard in school (dummies)	Yes	Yes	Yes
State dummies	Yes	Yes	Yes
Wald chi^2^	667.30	22885.08	1132.93
Number of observations	18,452	17,806	6,555

*Source:* Author’s computation based on data from IHDS-II (2011–12).

*Note:* The results are from the multi-level model, with children considered as first stage units and villages where the children reside as the second stage units. Estimates in Column III reflect Probit coefficients. Standard errors are clustered at the level of the primary sampling units (villages); reported in parentheses. *** p < 0.01, ** p < 0.05, * p < 0.1.

Caste differences are not significant in terms of the total study time, but we observe that educational expenditure is likely to highest for upper-caste Hindu families. In terms of the Math test score, as compared to the base outcome of an upper-caste Hindu family, the effect is again negative across other groups, but statistically significant amongst Scheduled Tribes (STs), Scheduled Castes (SCs), and Muslims. The level of development in a village influences educational expenses, with higher expenses in more developed villages, but the effect on other outcomes is not statistically significant at conventional levels.

Our key variable, that is, indoor access to piped water, is not statistically significant. Since the households with indoor access to piped water could be systematically different from those without such access, we do not comment on causality at this stage, and revisit this issue later when we carry out a set of robustness checks.

**Fig. 4:**
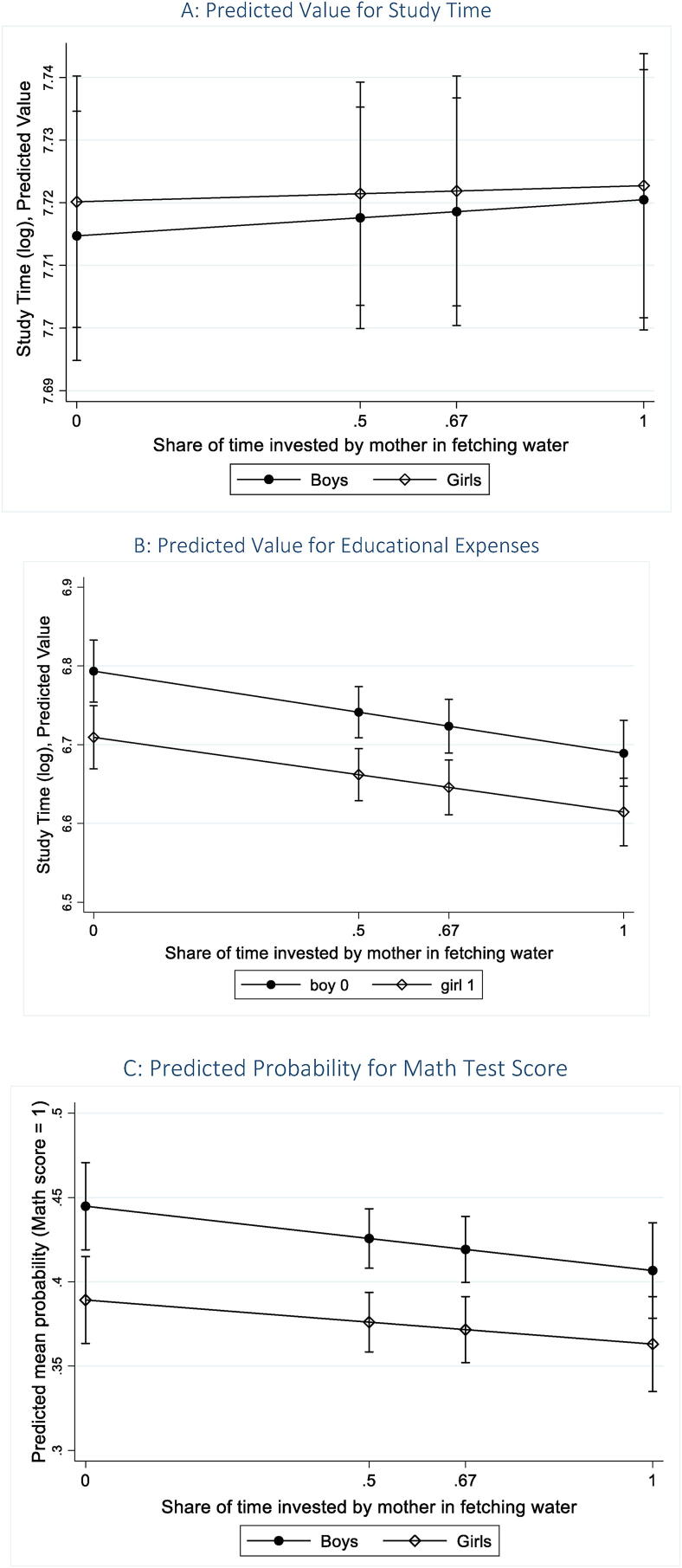
Effect of Mother’s Share of Time Invested in Fetching Water. *Source:* Authors’ computation based on data from IHDS-II (2011–12). *Notes:* Predicted value for children’s outcomes at different values of the ratio of the time invested by the mother to the total time invested by both the parents in fetching water (0, 25th percentile, 50th percentile, 75th percentile). The share values are 0, 0.5, 0.67, and 1 at the respective percentiles for households that fetch water. These predictive margins are based on estimates provided in Table 4A, Table 4B, using multi-level regressions.

For households without access to drinking water inside the premises, our findings suggest that an increase in the mother’s share of time spent in fetching water is negatively associated with both educational expenses and Math test score (see Table 4B). Interacting the mother’s share of the time invested in fetching water with the gender of the child, we observe that at each level (see Fig. 4A, 4B, and 4C) for the 25th, 50th, and 75th percentiles of the mother’s share of the time in unpaid work, even though all children suffer, girls are at a greater disadvantage. At the median value of 0.6, for households that fetch water from outside, the educational expenses on girls are lower by 8.3 percent than those for boys. But, an increase in the share of time invested leads to a greater decrease in educational outcomes for boys, though the magnitude of such an increase, is relatively small at 0.8 percent if the share of the mother’s unpaid work goes up by 0.1.

**Table 4B:** Marginal Effect of a Change in the Share of Mother’s Time in Fetching Water.

	Study Time	Educational Expenses	Math Score
	I	II	III
Boys	0.01	−0.10***	−0.04*
	(0.01)	(0.03)	(0.02)
Girls	0.00	−0.09***	−0.03
	(0.01)	(0.0)	(0.02)

*Source:* Author’s computation based on results from Table 4A, using data from IHDS-II (2011–12).

*Note:* The coefficients reflect the marginal effect of a change in the share of mother’s time in fetching water out of the total time invested by parents towards the task. Standard errors (in parentheses) have been calculated using the delta method. *** p < 0.01, ** p < 0.05, * p < 0.1.

In terms of the effect on the Math test score,^5^ we find that the predicted probability of girls being able to subtract or divide is lower than that of boys by 5 percentage points, statistically significant at the one percent level. In the absence of access to drinking water within household premises, at each level of the mother’s share of time in unpaid work, girls fare worse on the Math test—at the median value of 0.6, the marginal predicted mean of girls being able to subtract or divide is lower than that for boys by 5 percentage points. However, an increase in the mother’s share of time invested in fetching water reduces the predicted probability of being able to undertake rudimentary arithmetic operations by 3 percentage points for girls and by 4 percentage points by boys. This indicates that as the mother spends more time away from home in unpaid tasks, children’s cognitive outcome suffers, quite likely from the lack of supervision. But, the effect is statistically significant at the ten percent, and only for boys. Interestingly, we also find that children’s educational outcomes are positively associated with mother’s education, with the effect increasing at higher levels of mother’s education, which re-emphasizes the role mothers play in improving their children’s human capital potential.

## 6 Robustness checks

### 6.1 Entropy balancing

As a measure of robustness check, we employ the entropy balancing method, developed by Hainmueller (2012), to correct for covariate balance between our treated and control groups, with access to piped drinking water within the household premises serving as our binary treatment indicator. We allow our re-weighting scheme to adjust for covariate means, variances, and skewness, matching the control group with the treated group using the following set of variables: (a) child-level covariates, such as gender, grade in school; (b) school-level covariates, such as the type of school attended (private or government), distance to school in kilometers; (c) mother’s education (six categories); (d) household-level covariates, such as annual household income, whether the household uses electricity, caste and religion of the household, and the type of village that the household is situated in (developed or a less developed village) and state of residence; (e) village-level covariates: whether the village has piped water supply, distance to a paved road, distance to the nearest town, and distance to the residence of local Member of Legislative Assembly (MLA). The child, mother, and household-level covariates help us capture the broad spectrum of the socio-demographic characteristics of the households; the village-level covariates account for geographic correlates of infrastructure access, connectivity, and socio-political factors. Proximity to the local administrative representative is important since it can influence the installation of a public piped water distribution network within the village. Covariates used for balancing the distribution between our treatment and control groups are based on the literature on access to piped water, discussed in previous sections in this paper. Controlling for the range of covariates limits the possibility of the omitted variable bias that often plagues non-experimental work (Oster, 2019).

Table 1 in the Appendix provides details on the balancing, indicating the quality of matching achieved by using EB. The upper panel (1A) shows the sample distribution prior to matching, while the lower panel (1B) shows the post-matching distribution. The *t*-test examines the differences in means for each of the variables under consideration. A total of 3986 children are in the treated group, residing in households with access to indoor piped water, with another 16,205 children in the control group who do not have such access. Our results indicate that entropy balancing reduces the differences in the first three moments of each of the independent variables. Panel 1B presents the covariate distribution, achieved post-re-weighting, in terms of not just the means, but also variance and skewness.

We also calculate the percentage bias pre- and post-balancing, where the bias is measured as the percentage difference of the sample means between the treated and the control groups as a percentage of the square root of the mean of the sample variances in the treated and control groups (Rosenbaum & Rubin, 1985). Our results, presented in Tables 1A and 1B, show significant bias reduction for all the variables post-balancing, with EB ensuring that the desired covariate distribution is achieved for each of these covariates in the control group. For instance, before EB, 62 percent of the children in the treated group reside in more developed villages, and 40 percent of the children in the control group reside in more developed villages. After balancing, this proportion is 62 percent for both.

After the balancing exercise, we carry out OLS estimation (with standard errors clustered at the village level) of the effect of the treatment variable, indoor piped water connection, on the first two outcome variables, study time and educational expenses, followed by Probit estimation for the third outcome, Math test score. All these estimation methods use weights generated from EB, the results for which are presented in Table 5. We control for a set of observables used for entropy balancing: the type of school attended, distance to the school, standard in school, gender of the child, presence of electricity in the household, mother’s education, caste and religion group, household income, and development of the village. We also include state fixed effects to account for the possibility of unobserved heterogeneity across states and differences in state-level policy initiatives.

**Table 5:** Outcome Means for Treated and Non-treated Households, along with the Marginal Effect of Treatment for Indoor Piped Water Connection.

			Treated	Non-treated	Marginal Effect	N
			(1)	(2)	(1) – (2)	
I	Study Time (log)	Girls	7.78***	7.75***	0.03	18,452
			(0.02)	(0.02)	(0.02)	
		Boys	7.75***	7.77***	−0.01	
			(0.02)	(0.02)	(0.02)	
II	Educational Expenses (log)	Girls	7.36***	7.26***	0.10**	17,806
			(0.03)	(0.03)	(0.04)	
		Boys	7.39***	7.36***	0.03	
			(0.03)	(0.03)	(0.04)	
III	Probability (Math Score = 1)	Girls	0.55***	0.57***	−0.01	6,555
			(0.02)	(0.02)	(0.03)	
		Boys	0.60***	0.57***	0.03	
			(0.02)	(0.02)	(0.03)	

*Source:* Author’s computation, using data from IHDS-II (2011–12).

*Note:* Models I and II are estimates from OLS regressions, with robust standard errors. Model III reflects estimates from Probit regression predicting the score on the Math test. Estimates are based on weights obtained from entropy balancing. Standard errors have been calculated using the delta method. The other control variables are the type of school attended, distance to school, standard in school, gender of the child, presence of electricity in the household, mother’s education, caste and religion group, household income, level of development of the village, and State fixed effects. *** p < 0.01, ** p < 0.05, * p < 0.1.

**Fig. 5:**
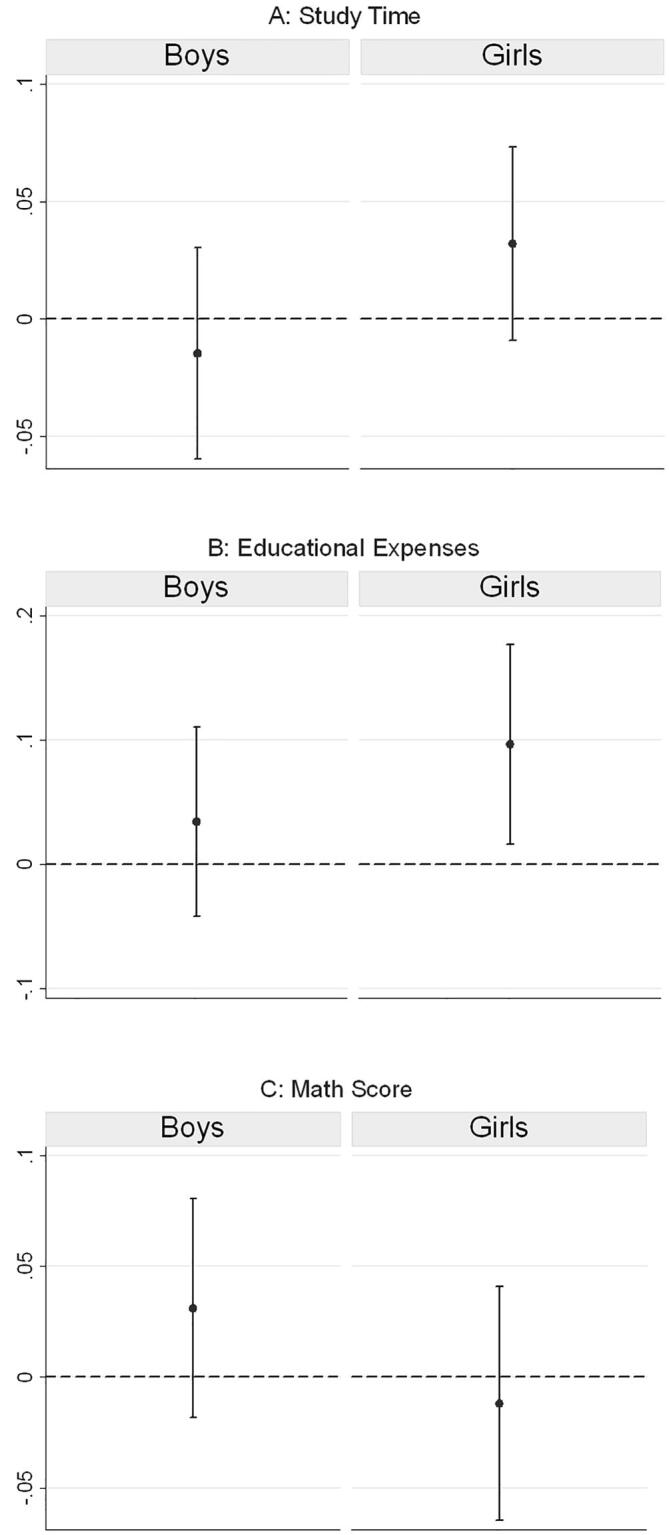
Marginal Effect of Indoor Access to Piped Water (Treatment) on Schooling Outcomes. *Source:* Authors’ computation based on data from IHDS-II (2011–12). *Notes:* The marginal effect of indoor access to a piped water connection (the treatment variable) on children’s outcomes is based on results from Table 5. Regression adjusted for weights from entropy balancing, which matched the covariate distribution of the non-treated group to the treated group based on the first three moments: mean, variance, and skewness.

Comparing households that have indoor piped water connections to those that do not (see Fig. 5A, 5B, and 5C), we find that the presence of a piped water connection within the household premises does not affect the study.^6^ In terms of the annual educational expenditure, we find a statistically significant increase in educational outlay by 10 percent for girls. The predicted probability of scoring in the Math test score is not statistically significant for either boys or girls at the conventional levels^7^.

## 7 Discussion and implications

Much of the research association of maternal inputs into children’s human capital development has focused on mother’s paid work activities, with limited evidence on the effect of unpaid work driven by inadequate access to infrastructure. Such unpaid work is more pervasive in rural areas, where households have to deal with the twin challenge of insufficient access to clean fuel and an inadequate piped water network, resulting in a heavy dependence on common resources, with women typically bearing a greater burden of the drudgery of such work.

Our findings suggest that children in households which do not spend time collecting firewood and water have substantially higher educational outcomes. Moreover, the negative impacts are greater when gender inequality in the household division of labor leads to women bearing a disproportionate burden of these unpaid activities. Thus, both the overall unpaid work burden as well as the concentration of this burden for women have significant negative associations with child outcomes.

Increasing the share of mothers in unpaid work, in the event of lack of adequate access to time-saving infrastructure, may lead to substitution of time away from childcare and related activities. Interestingly enough, while girls face a greater disadvantage as compared to boys across all the three outcome variables, our findings from the analysis on fuelwood collection suggest that, as the mother’s share of the unpaid work increases, the predicted values of study time and educational expenses are lesser for boys. In terms of the Math test score, the marginal effect is negative for both, but statistically significant only for boys (at the ten percent level), indicating the possibility of school-going boys suffering more than girls, potentially from lack of supervision by the mother. While this does not support our expectation (Hypothesis H3), it is consistent with the literature in developed countries, which suggest greater psychosocial fragility for boys, resulting in greater risks posed by maternal employment for boys (Brooks-Gunn, Han, & Waldfogel, 2010).

We find similar results for households without access to piped water (see Table 4B), for children’s educational expenses and Math test score, emphasizing that mother’s unpaid work away from home is detrimental for children. Not surprisingly, mother’s education is positively associated with children’s outcome, suggesting the pivotal role mothers play in children’s educational outcomes.

In the light of the evidence suggesting the increased relevance of maternal inputs in the context of at-home learning requirements, an issue that has come to the forefront in the midst of the COVID-19 pandemic-induced lockdown, it is important to the public policy discourse to develop time-saving technologies that can reduce the acute time poverty that many women face. However, access to time-saving technologies also needs to be cost-effective for rural families, who often switch to common resources, when energy prices go up, as is evident from our first stage results predicting the likelihood of households collecting fuelwood. Thus, it is important to enhance access to energy markets in rural areas, not only in terms of physical access via an improved distribution network, and transportation systems and a broader network of paved roads, but also in terms of providing adequate financial assistance. The coverage and distribution network has expanded rapidly in recent years, improving equity in clean energy access. While government schemes such as Ujjawal Yojana have increased access to LPG gas, recent studies (Gould & Urpelainen, 2019) show a poor uptake of LPG refills, primarily driven by cost considerations. Redesigning the LPG pricing scheme with higher subsidies for households below the poverty line, may induce switching away from biofuel usage and greater adoption of LPG, moving India closer to realization of Sustainable Development Goal (SDG) 7, which seeks to provide access to affordable, reliable, sustainable, and clean energy for all. For instance, (Smith & Harish, 2019) noted that targeted support for vulnerable groups, along with making a rural LPG distribution network more viable can increase the usage of clean fuel.

Incidentally, the continued practice of fuel stacking in households across all income quintiles, especially in rural areas, also indicates that it is important to simultaneously reinforce the message on the benefits of adopting clean fuel such as LPG, incorporating the gender dimension and intergenerational transmission into such public policy discourse. This becomes all the more important, given our findings that the mother’s time away from home, invested in low-productive unpaid work, is detrimental to children’s wellbeing and educational outcomes. Evidence shows that women’s access to financial resources, and more importantly control over such resources, improves clean fuel adoption, (Choudhuri and Desai, 2020, Das et al., 2020, Gould and Urpelainen, 2019), emphasizing the synergies between the realization of access to sustainable energy sources and achieving gender equality, which has also guided public policies in India in recent years.

Along with aiding rural families to meet their energy needs, it is pertinent to improve the village water distribution network to alleviate the burden it imposes on women’s time endowment. Such time constraints get further exacerbated in the summer months or drought years, especially in regions that face an acute water crisis, with women typically facing the brunt of such adversarial conditions. More recently, the government has launched a nationwide program to ensure access to safe drinking water for all households in both rural and urban areas by the year 2024. Such initiatives have the potential to generate significant time savings, particularly for women, who spend considerable time each day towards fetching water, which reduces the time available for other productive work. The importance of developing programs to reduce gender disparities in access to resources (Agarwal, 2001) becomes all the more critical in the light of findings in the current paper, especially in disentangling any adverse channel of intergenerational transmission between the mother and her children.

It is also important to note that while the overall work burden involved in the free collection of water and firewood has negative implications for children, gender inequality in its distribution plays an important and independent role. Children living in households in which women bear a disproportionate share of this burden are disadvantaged in terms of educational expenditure, study time, and mathematical skills. This suggests that in addition to overall reduction in the work burden, a more equitable distribution of the unpaid work burden may also be associated with child outcomes, an area of research that deserves further attention.

## CRediT authorship contribution statement

**Pallavi Choudhuri:** Conceptualization, Data curation, Formal analysis, Methodology, Software, Validation, Visualization, Writing – original draft, Writing – review & editing. **Sonalde Desai:** Conceptualization, Data curation, Funding acquisition, Investigation, Methodology, Project administration, Resources, Software, Supervision, Validation, Visualization, Writing – original draft, Writing – review & editing

## Declaration of Competing Interest

The authors declare that they have no known competing financial interests or personal relationships that could have appeared to influence the work reported in this paper.

## Figures and Tables

**Table 1:** Entropy Balancing for First Three Moments for Households with and without Indoor Access to Piped Water.

	Panel 1A: Before Weighting							
Variables	Treated Units: 3930			Control Units: 16,196				
	Mean	Variance	Skewness	Mean	Variance	Skewness	% bias	t-test
School (private = 1)	0.66	0.22	−0.68	0.72	0.20	−1.00	−13.50	−7.71
Omitted: govt. = 0								
Distance to school	2.56	12.99	4.30	2.04	11.97	10.28	14.70	8.35
Gender (female = 1)	0.47	0.25	0.14	0.48	0.25	0.10	−2.20	−1.21
Omitted: male = 0								
Standard (std.) in school								
<1 std.	Omitted							
1st std.	0.06	0.06	3.62	0.10	0.09	2.72	−12.90	−6.85
2nd std.	0.10	0.09	2.59	0.13	0.11	2.26	−6.60	−3.65
3rd std.	0.12	0.11	2.29	0.13	0.11	2.22	−1.70	−0.95
4th std.	0.11	0.09	2.57	0.12	0.10	2.38	−3.80	−2.11
5th std.	0.12	0.10	2.37	0.12	0.11	2.33	−0.90	−0.48
6th std.	0.12	0.10	2.38	0.11	0.10	2.53	3.00	1.71
7th std.	0.11	0.10	2.49	0.10	0.09	2.70	4.00	2.26
8th std.	0.11	0.10	2.48	0.08	0.08	3.01	9.10	5.34
9th std.	0.12	0.11	2.31	0.08	0.08	3.00	12.50	7.42
Mother's education								
Illiterate	omitted							
1–4 std.	0.19	0.15	1.62	0.17	0.14	1.77	4.40	2.49
5–9 std.	0.24	0.18	1.19	0.18	0.15	1.67	16.00	9.33
10–11 std.	0.14	0.12	2.05	0.06	0.06	3.69	27.30	17.42
12th and some college	0.08	0.07	3.08	0.04	0.04	4.71	17.10	10.75
Graduate and above	0.05	0.05	4.14	0.03	0.03	5.94	12.40	7.72
Caste and Religion								
Forward Caste Hindus	omitted							
OBC	0.34	0.01	0.69	0.35	0.00	0.61	−3.40	−1.90
Scheduled Caste	0.26	0.19	1.12	0.23	0.18	1.29	6.40	3.63
Scheduled Tribe	0.06	0.06	3.70	0.11	0.09	2.57	−16.40	−8.58
Muslim	0.08	0.08	3.03	0.15	0.13	1.99	−20.50	−10.75
Christian, Sikh, Jain	0.02	0.02	6.24	0.01	0.01	8.63	8.20	5.09
States/UTs								
Jammu & Kashmir	omitted							
Himachal Pradesh	0.11	0.10	2.49	0.03	0.03	5.54	31.8	21.39
Uttarakhand	0.01	0.01	8.69	0.02	0.02	7.16	−4.6	−2.44
Punjab	0.09	0.09	2.77	0.04	0.04	4.82	22.8	14.45
Haryana	0.16	0.14	1.84	0.05	0.05	4.22	37.6	24.75
Uttar Pradesh	0.01	0.01	9.18	0.22	0.17	1.39	−67.8	−30.78
Jharkhand	0.01	0.01	11.31	0.03	0.03	5.23	−18.0	−8.62
Rajasthan	0.09	0.08	2.91	0.10	0.09	2.63	−4.8	−2.63
Chhattisgarh	0.01	0.01	8.88	0.06	0.06	3.65	−26.4	−12.52
Madhya Pradesh	0.02	0.02	6.27	0.15	0.13	1.98	−45.6	−21.48
North-east	0.03	0.03	5.56	0.01	0.01	9.36	13.1	8.44
West Bengal	0.01	0.01	11.12	0.06	0.06	3.62	−29.9	−13.87
Gujarat	0.09	0.08	2.93	0.03	0.03	5.62	24.9	16.22
Maharashtra and Goa	0.15	0.13	1.91	0.07	0.06	3.47	28.6	17.85
Andhra Pradesh	0.05	0.04	4.29	0.03	0.03	5.22	7.0	4.10
Karnataka	0.09	0.08	2.95	0.06	0.05	3.86	11.8	6.95
Kerala	0.01	0.01	13.91	0.02	0.02	7.08	−12.8	−6.16
Tamil Nadu	0.02	0.02	6.45	0.01	0.01	8.69	7.3	4.45
Household covariates								
Household has electricity	0.98	0.00	−6.94	0.76	0.00	−1.20	70.00	32.24
Income (log)	11.44	0.96	0.07	10.99	0.94	−0.15	46.00	25.91
Village-level covariates								
Piped water source	0.92	0.00	−3.22	0.39	0.00	0.47	137.70	67.14
Distance to paved roads	0.79	14.72	7.47	0.52	4.47	7.10	8.70	5.93
Distance to the nearest town	12.19	87.99	1.58	13.33	126.80	2.47	−11.00	−5.90
Distance to the local MLA	2.94	0.11	−5.29	2.89	0.18	−3.83	12.30	6.47
Sector (village)								
Less developed	omitted							
More developed	0.62	0.01	0.50	0.40	0.00	−0.39	44.50	24.94

	Panel B: After Weighting							
Variables	Treated Units: 3930			Control Units: 16,196				
	Mean	Variance	Skewness	Mean	Variance	Skewness	% bias	*t*-test
School (private = 1)	0.66	0.22	−0.68	0.66	0.22	−0.68	0.10	0.03
Omitted: govt. = 0								
Distance to school	2.56	12.99	4.30	2.56	13.01	4.31	0.00	0.01
Gender (female = 1)	0.47	0.25	0.14	0.47	0.25	0.14	0.00	−0.02
Omitted: male = 0								
Standard (std.) in school								
<1 std.	0.06	0.06	3.62	0.06	0.06	3.62	0.00	−0.02
1st std.	0.10	0.09	2.58	0.10	0.09	2.58	−0.10	−0.03
2nd std.	0.12	0.11	2.29	0.12	0.11	2.29	−0.10	−0.03
3rd std.	0.11	0.09	2.58	0.11	0.09	2.58	0.00	0.01
4th std.	0.12	0.10	2.37	0.12	0.10	2.37	−0.10	−0.03
5th std.	0.12	0.10	2.38	0.12	0.10	2.38	0.00	0.01
6th std.	0.11	0.10	2.49	0.11	0.10	2.49	0.10	0.04
7th std.	0.11	0.10	2.48	0.11	0.10	2.48	0.00	0.01
8th std.	0.12	0.11	2.31	0.12	0.11	2.31	0.00	0.00
9th std.	0.06	0.06	3.62	0.06	0.06	3.62	0.00	−0.02
Mother's education								
Illiterate	Omitted							
1–4 std.	0.19	0.15	1.61	0.19	0.15	1.61	−0.10	−0.04
5–9 std.	0.24	0.18	1.19	0.24	0.18	1.19	0.10	0.03
10–11 std.	0.14	0.12	2.05	0.14	0.12	2.05	−0.10	−0.03
12th and some college	0.08	0.07	3.09	0.08	0.07	3.09	0.10	0.02
Graduate and above	0.05	0.05	4.14	0.05	0.05	4.14	0.00	−0.02
Caste and Religion								
Forward Caste Hindus	0.34	0.22	0.69	0.34	0.22	0.69	0.00	−0.01
OBC	0.26	0.19	1.11	0.26	0.19	1.11	−0.10	−0.05
Scheduled Caste	0.06	0.06	3.69	0.06	0.06	3.69	0.00	−0.02
Scheduled Tribe	0.08	0.08	3.03	0.08	0.08	3.03	0.00	−0.02
Muslim	0.02	0.02	6.27	0.02	0.02	6.27	0.20	0.06
Christian, Sikh, Jain	0.34	0.22	0.69	0.34	0.22	0.69	0.00	−0.01
States/UTs								
Jammu & Kashmir	Omitted							
Himachal Pradesh	0.11	0.10	2.49	0.11	0.10	2.49	−0.1	−0.03
Uttarakhand	0.01	0.01	8.69	0.01	0.01	8.69	−0.0	−0.01
Punjab	0.09	0.09	2.77	0.09	0.09	2.77	−0.1	−0.02
Haryana	0.16	0.14	1.84	0.16	0.14	1.84	−0.1	−0.03
Uttar Pradesh	0.01	0.01	9.18	0.01	0.01	9.18	−0.0	−0.01
Jharkhand	0.01	0.01	11.31	0.01	0.01	11.31	−0.0	−0.01
Rajasthan	0.09	0.08	2.91	0.09	0.08	2.91	−0.0	−0.02
Chhattisgarh	0.01	0.01	8.88	0.01	0.01	8.88	−0.0	−0.01
Madhya Pradesh	0.02	0.02	6.27	0.02	0.02	6.27	−0.0	−0.01
North-east	0.03	0.03	5.56	0.03	0.03	5.56	−0.0	−0.01
West Bengal	0.01	0.01	11.12	0.01	0.01	11.12	−0.0	−0.01
Gujarat	0.09	0.08	2.93	0.09	0.08	2.93	−0.1	−0.02
Maharashtra and Goa	0.15	0.13	1.91	0.15	0.13	1.91	0.4	0.15
Andhra Pradesh	0.05	0.04	4.29	0.05	0.04	4.29	−0.0	−0.02
Karnataka	0.09	0.08	2.95	0.09	0.08	2.95	−0.1	−0.02
Kerala	0.01	0.01	13.91	0.01	0.01	13.91	−0.0	−0.01
Tamil Nadu	0.02	0.02	6.45	0.02	0.02	6.45	−0.0	−0.01
Household covariates								
Household has electricity	0.98	0.02	−6.93	0.98	0.02	−6.93	0.00	0.01
Income (log)	11.44	0.96	0.07	11.44	0.96	0.07	0.00	0.01
Village-level covariates								
Piped water source	0.92	0.07	−3.21	0.92	0.07	−3.21	0.00	0.02
Distance to paved roads	0.79	14.72	7.47	0.79	14.71	7.47	0.00	−0.01
Distance to the nearest town	12.19	87.99	1.58	12.19	87.99	1.58	0.00	0.01
Distance to the local MLA	2.94	0.11	−5.29	2.94	0.11	−5.29	−0.20	−0.08
Sector (village)								
Less developed	Omitted							
More developed	0.62	0.24	−0.50	0.62	0.24	−0.50	0.10	0.06

## Footnotes

1 The energy demand for lighting is met by either kerosene or electricity, and does not induce a time burden on households in terms of collection time or search time, as in the case of firewood.

2 For the survey description, see https://www.ihds.umd.edu/home. The IHDS data from wave II are available for download from the ICPSR website: https://www.icpsr.umich.edu/web/DSDR/studies/36151. Files *DS1: Individual, DS2: Household, and DS12: Village* have been used for this study.

3 These estimates are calculated based on first stage results for the regression model for column I, with logarithm of study time as the outcome variable. The estimates from other regression models are consistent and robust.

4 The ratio of one indicates that the task of collecting fuelwood is borne out entirely by the mother.

5 We also examined the impact of lack of access to piped water and clean fuel on children’s reading assessments. Most of the impacts were in the same direction, with some effects significant at the 0.05 level and others at the 0.1 level. It is not surprising to have stronger impacts on mathematical achievement. Meta-analysis of studies that have looked at the impact of socioeconomic conditions on student achievement in developed countries have usually found greater impact on mathematical achievement than on reading scores (Sirin, 2005; Cooper, Nye, Charlton, Lindsay, & Greathouse, 1996). Recent data on the impact of COVID-related school shutdown and increased parental responsibility for children’s education also shows that the shutdown has affected mathematical scores, leaving reading scores unchanged (Kuhfeld et al., 2020).

6 Note that the estimates for marginal effects in Table 4 reflect the effect of access to a piped water connection on the logarithm of the study time and educational expenses. For our discussions in this section, we have presented the exponentiated estimates.

7 As an additional robustness check, we replace annual household income with household assets across all sets of regressions (for fuelwood collection and piped water connection), constructing an asset index from a set of 31 basic and durable goods owned by households. We leave out access to indoor piped water connection and LPG from assets index to avoid confoundedness. Most of the regression estimates were in the same direction, with some effects significant at the five percent level and others at the ten percent level. The lower level of significance in some cases could be because current income may be a better indicator of contemporaneous budget allocation towards household expenses, including educational expenses, and hence has a direct impact on educational outcomes. The detailed estimation results controlling for household assets are available on request.

## References

[b0005] Agarwal B. (1997). “Bargaining” and gender relations: Within and beyond the household. Feminist Economics.

[b0010] Agarwal B. (2001). Participatory exclusions, community forestry, and gender: An analysis for South Asia and a conceptual framework. World Development.

[b0015] Amacher G.S., Hyde W.F., Kanel K.R. (1996). Household fuelwood demand and supply in Nepal's tarai and mid-hills: Choice between cash outlays and labor opportunity. World Development.

[b0020] Balakrishnan K., Ramaswamy P., Sambandam S., Thangavel G., Ghosh S., Johnson P., Thanasekaraan V. (2011). Air pollution from household solid fuel combustion in India: An overview of exposure and health related information to inform health research priorities. Global Health Action.

[b0025] Bank W. (2011). World development report 2012: Gender equality and development.

[b0030] Bassani D.G., Jha P., Dhingra N., Kumar R. (2010). Child mortality from solid-fuel use in India: A nationally-representative case-control study. BMC Public Health.

[b0035] Becker G. (1981). A treatise on the family.

[b0040] Becker G.S. (1993). A treatise on the family.

[b0045] Bernal R. (2008). The effect of maternal employment and child care on children's cognitive development. International Economic Review.

[b0050] Bianchi S.M. (2000). Maternal employment and time with children: Dramatic change or surprising continuity?. Demography.

[b0055] Brooks-Gunn J., Han W.-J., Waldfogel J. (2010). First-year maternal employment and child development in the first 7 years. Monographs of the Society for Research in Child Development.

[b0060] Carneiro P., Løken K.V., Salvanes K.G. (2015). A flying start? Maternity leave benefits and long-run outcomes of children. Journal of Political Economy.

[b0065] Chafe Z.A., Brauer M., Klimont Z., Van Dingenen R., Mehta S., Rao S., Smith K.R. (2014). Household cooking with solid fuels contributes to ambient PM2.5 air pollution and the burden of disease. Environmental Health Perspectives.

[b0070] Choudhuri P., Desai S. (2020). Gender inequalities and household fuel choice in India. Journal of Cleaner Production.

[b0075] Cooper H., Nye B., Charlton K., Lindsay J., Greathouse S. (1996). The effects of summer vacation on achievement test scores: A narrative and meta-analytic review. Review of Educational Research.

[b0080] Crow B., Mcpike J. (2009). How the drudgery of getting water shapes women’s lives in low-income urban communities. Gender, Technology and Development.

[b0085] Currie J., Stabile M., Manivong P., Roos L.L. (2010). Child health and young adult outcomes. Journal of Human Resources.

[b0090] Das, I., Klug, T., Krishnapriya, P., Plutshack, V., Saparapa, R., Scott, S., . . . Pattanayak, S. K. (2020). A Virtuous Cycle? Reviewing the evidence on women’s empowerment and energy access, frameworks, metrics and methods. Retrieved from https://energyaccess.duke.edu/wp-content/uploads/2020/03/White-paper-on-gender-and-energy-access-1.pdf.

[b0095] Desai S., Chase-Lansdale P.L., Michael R.T. (1989). Mother or market? Effects of maternal employment on the intellectual ability of 4-year-old children. Demography.

[b0100] Desai S., Dubey A., Vanneman R., Banerji R. (2009). Private schooling in India: A new educational landscape. Paper presented at the India Policy Forum.

[b0105] Desai S., Jain D. (1994). Maternal employment and changes in family dynamics: The social context of women's work in rural South India. Population and Development Review.

[b0110] Desai, S., & Vanneman, R. (2018). India human development survey-II (IHDS-II), 2011-12.

[b0115] Devoto F., Duflo E., Dupas P., Parienté W., Pons V. (2012). Happiness on tap: Piped water adoption in urban Morocco. American Economic Journal: Economic Policy.

[b0120] Doepke M., Tertilt M. (2011). Does female empowerment promote economic development?.

[b0125] Doss C. (2013). Intrahousehold bargaining and resource allocation in developing countries. The World Bank Research Observer.

[b0130] Flavio C., James J.H. (2009). Investing in our young people. Rivista Internazionale di Scienze Sociali.

[b0135] Geruso M., Spears D. (2018). Neighborhood sanitation and infant mortality. American Economic Journal: Applied Economics.

[b0140] Goldstein, H. (2011). Multilevel statistical models (Vol. 922): John Wiley & Sons.

[b0145] Gould C.F., Urpelainen J. (2019). The gendered nature of liquefied petroleum gas stove adoption and use in rural India. The Journal of Development Studies.

[b0150] Gupta A. (2019). Where there is smoke: Solid fuel externalities, gender, and adult respiratory health in India. Population and Environment.

[b0155] Hainmueller J. (2012). Entropy balancing for causal effects: A multivariate reweighting method to produce balanced samples in observational studies. Political Analysis.

[b0160] Heckman, J., & Carneiro, P. (2003). Human Capital Policy. Retrieved from https://EconPapers.repec.org/RePEc:nbr:nberwo:9495.

[b0165] Heckman, J. J. (1976). The common structure of statistical models of truncation, sample selection and limited dependent variables and a simple estimator for such models. In Annals of economic and social measurement, volume 5, number 4 (pp. 475-492): NBER.

[b0170] Heckman J., Stixrud J., Urzua S. (2006). The effects of cognitive and noncognitive abilities on labor market outcomes and social behavior. Journal of Labor Economics.

[b0175] Hill J.L., Waldfogel J., Brooks-Gunn J., Han W.-J. (2005). Maternal employment and child development: A fresh look using newer methods. Developmental Psychology.

[b0180] Hirway I., Jose S. (2011). Understanding women's work using time-use statistics: The case of India. Feminist Economics.

[b0185] Ilahi N., Grimard F. (2000). Public infrastructure and private costs: Water supply and time allocation of women in rural Pakistan. Economic Development and Cultural Change.

[b0190] Jayachandran S. (2015). The roots of gender inequality in developing countries. Annual Review of Economics.

[b0195] Kabeer N. (2012). Women’s economic empowerment and inclusive growth: Labour markets and enterprise development. International Development Research Centre.

[b0200] Köhlin G., Sills E.O., Pattanayak S.K., Wilfong C. (2011). Energy, gender and development: What are the linkages? Where is the evidence?.

[b0205] Kookana R.S., Maheshwari B., Dillon P., Dave S.H., Soni P., Bohra H., Patel A. (2016). Groundwater scarcity impact on inclusiveness and women empowerment: Insights from school absenteeism of female students in two watersheds in India. International Journal of Inclusive Education.

[b0210] Koolwal G., van de Walle D. (2013). Access to water, women’s work, and child outcomes. Economic Development and Cultural Change.

[b0215] Kuhfeld M., Soland J., Tarasawa B., Johnson A., Ruzek E., Liu J. (2020). Projecting the potential impact of COVID-19 school closures on academic achievement. Educational Researcher.

[b0220] Leibowitz A. (1974). Home investments in children. Journal of Political Economy.

[b0225] Lewis J.J., Pattanayak S.K. (2012). Who adopts improved fuels and cookstoves? A systematic review. Environmental Health Perspectives.

[b0230] Lundberg S.J., Pollak R.A., Wales T.J. (1997). Do husbands and wives pool their resources? Evidence from the United Kingdom child benefit. Journal of Human Resources.

[b0235] Mangyo E. (2008). The effect of water accessibility on child health in China. Journal of Health Economics.

[b0240] Meeks R.C. (2017). Water Works: The economic impact of water infrastructure. Journal of Human Resources.

[b0245] Menon N., van der Meulen Rodgers Y., Nguyen H. (2014). Women’s land rights and children’s human capital in Vietnam. World Development.

[b0250] Muralidharan K., Kremer M. (2008). Public and private schools in rural India.

[b0255] Narain U., Gupta S., van 't Veld K. (2008). Poverty and resource dependence in rural India. Ecological Economics.

[b0260] Oster E. (2019). Unobservable selection and coefficient stability: Theory and evidence. Journal of Business & Economic Statistics.

[b0265] Raudenbush, S. W., & Bryk, A. S. (2002). Hierarchical linear models: Applications and data analysis methods (Vol. 1): sage.

[b0270] Jalan J., Ravallion M. (2003). Does piped water reduce diarrhea for children in rural India?. Journal of Econometrics.

[b0275] Ray I. (2007). Women, water, and development. Annual Review of Environment and Resources.

[b0280] Rosenbaum P.R., Rubin D.B. (1985). Constructing a control group using multivariate matched sampling methods that incorporate the propensity score. The American Statistician.

[b0285] Ruhm C.J. (2008). Maternal employment and adolescent development. Labour Economics.

[b0290] Sayer L., Bianchi S., Robinson J. (2004). Are parents investing less in children? Trends in mothers’ and fathers’ time with children. American Journal of Sociology.

[b0295] Seiz J.A. (1995). Bargaining models, feminism, and institutionalism. Journal of Economic Issues.

[b0300] Sirin S.R. (2005). Socioeconomic status and academic achievement: A meta-analytic review of research. Review of Educational Research.

[b0305] Smith, K. R., & Harish, S. (2019). Ujjwala 2.0: From Access to Sustained Usage (Vol. CCAPC/2019/03). Collaborative Clean Air Policy Centre (CCAPC), New Delhi.

[b0310] Spears D. (2012). Height and cognitive achievement among Indian children. Economics & Human Biology.

[b0315] Vikram K., Chen F., Desai S. (2018). Mothers' work patterns and Children's cognitive achievement: Evidence from the India Human Development survey. Social Science Research.

[b0320] Woolley F.R. (1993). The feminist challenge to neoclassical economics. Cambridge Journal of Economics.

